# Angiosarcoma of the breast with hypofibrinogenemia: A rare case report and review of the literature

**DOI:** 10.3389/fonc.2022.1047935

**Published:** 2022-11-02

**Authors:** Ran An, Jing-Yi Ma, Xi-Hao Ni, Chang-Liang Wang

**Affiliations:** ^1^ School of Clinical Medicine, Weifang Medical University, Weifang, China; ^2^ Department of Breast Surgery, Weifang People’s Hospital, Weifang, Shandong, China

**Keywords:** angiosarcoma, breast, hypofibrinogenemia, case report, review of the literature

## Abstract

**Background:**

Breast angiosarcoma is a rare malignant tumor, accounting for approximately 0.04% of all breast malignancies. Angiosarcoma of the breast with hypofibrinogenemia is even rarer and has not been described in man. Breast angiosarcoma is associated with high metastatic potential and poor prognosis, and there is no specific manifestation in imaging. At present, surgery is considered to be the only effective treatment. There is no unified standard for postoperative adjuvant radiotherapy and chemotherapy.

**Case Presentation:**

A 30-year-old female patient underwent left breast mass resection under local anesthesia on May 22, 2014. Postoperative pathology showed a vasogenic tumor. On November 10, 2017, she visited us again due to left breast swelling and pain during lactation, and underwent breast mass puncture. She was diagnosed with breast hematoma and fibrinogen reduction. On November 14, 2017, mastectomy was performed under tracheal intubation and general anesthesia, and the fibrinogen gradually returned to normal after surgery. Pathological examination showed a hemangiosarcoma with hematoma formation in the left breast. According to the pathological findings and after comprehensive evaluation, the patient underwent modified radical mastectomy for left breast cancer and right axillary sentinel lymph node biopsy on December 5, 2017. The patient died on January 28, 2018 due to rupture and hemorrhage of liver cancer and hemorrhagic shock.

**Conclusion:**

Breast angiosarcoma with hypofibrinogenemia is a rare and highly aggressive malignancy. Clinicians should be familiar with its clinicopathological features and diagnostic criteria. Multidisciplinary approach is recommended to benefit the patients.

## Introduction

Breast angiosarcoma is a rare malignant breast tumor that originates in perilobular or lobular capillary endothelium, accounting for approximately 0.04% of all breast malignancies (1). Angiosarcoma of the breast with hypofibrinogenemia is even more infrequent. Hypofibrinogenemia is defined as plasma fibrinogen concentration < 2 g/L. There are many causes of the disease, and the present case may have been caused by acute bleeding or related to the individual’s physical condition.

We retrospectively reviewed the medical records of one case of breast angiosarcoma with hypofibrinogenemia admitted to Weifang People’s Hospital, together with a review of the literature, to analyze the diagnosis and treatment of breast angiosarcoma, to improve awareness of breast angiosarcoma and its secondary diseases, and thus reduce misdiagnosis or mistreatment.

## History

**Figure 1:**
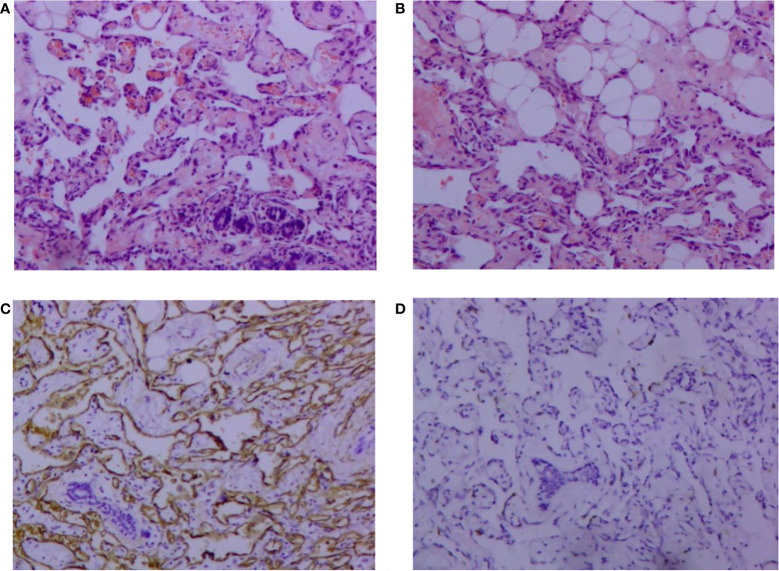
Pathological findings of vascular neoplasms of the breast (hematoxylin and eosin, 100×). **(A)** Tumor cells infiltrating breast tissue and invading lobule tissue; **(B)** Tumor cells invading adipose tissue. Immunohistochemical image of vascular neoplasms of the breast (SP 100×); **(C)** positive immunohistochemical staining for CD34; **(D)**: positive immunohistochemical staining of Ki-67.

A 30-year-old female patient was admitted to the Breast Surgery Department of Weifang People’s Hospital on May 21, 2014 because of a left breast mass for > 1 year. On clinical examination, there was a palpable mass about the size of a peanut, with slight tenderness and no local skin redness and swelling. Color Doppler ultrasonography showed that the lower inner quadrant had a low echo of 1.2 cm × 0.7cm in the left breast, with unclear boundary and tendency to hyperplasia. After comprehensive consideration, left breast mass resection was performed under local anesthesia on May 22, 2014. Postoperative pathology showed: vasogenic tumor; In view of the special location of the tumor, angiosarcoma was not excluded (**Figures 1A, B**). Immunohistochemistry showed: vimentin (+), CD31 (+), CD34 (+) and Ki67 (10%) (**Figures 1C, D**). We recommended close follow-up.

**Figure 2:**
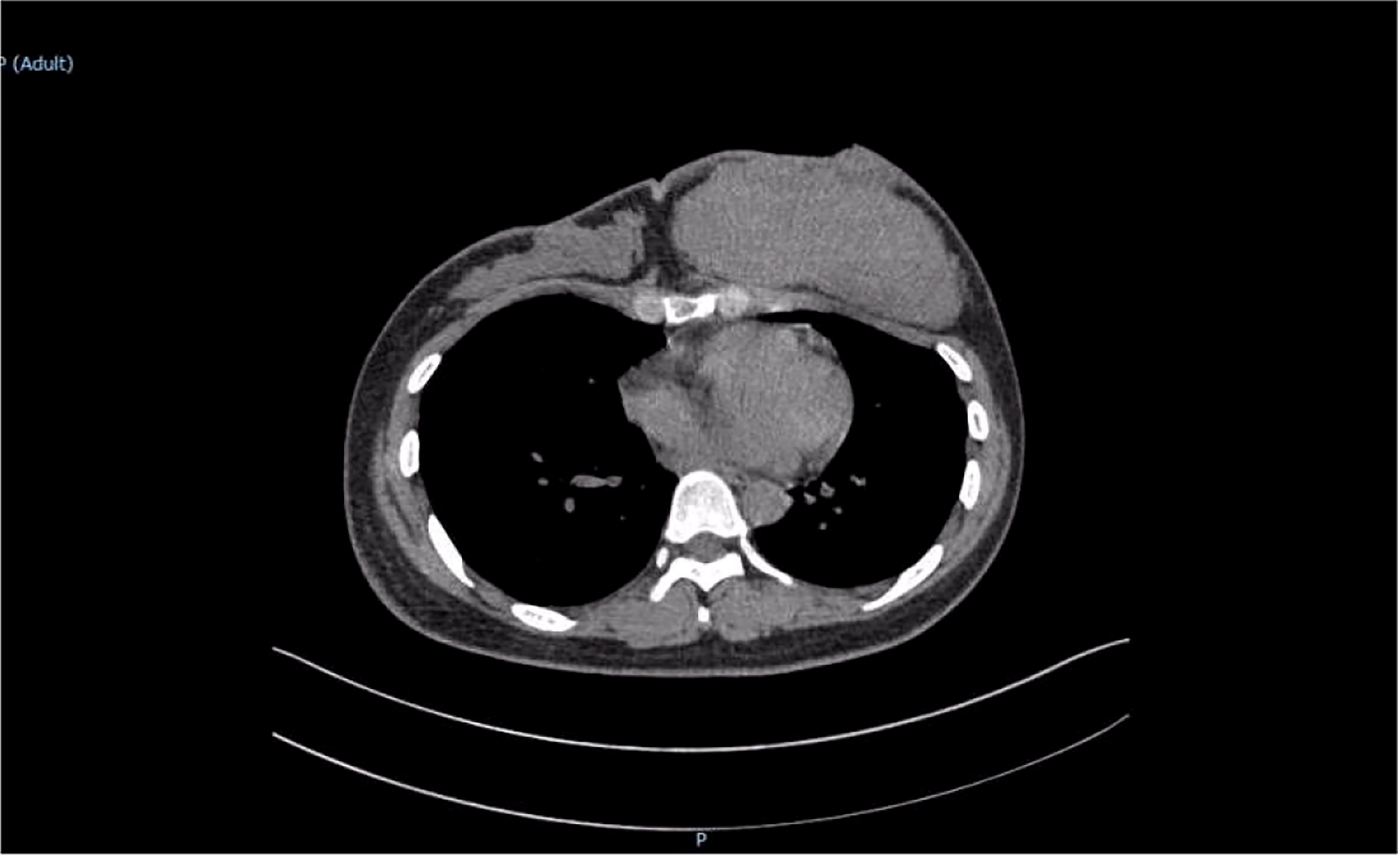
Chest computed tomography scan revealed a large soft tissue mass in the left breast with uniform density.

**Figure 3:**
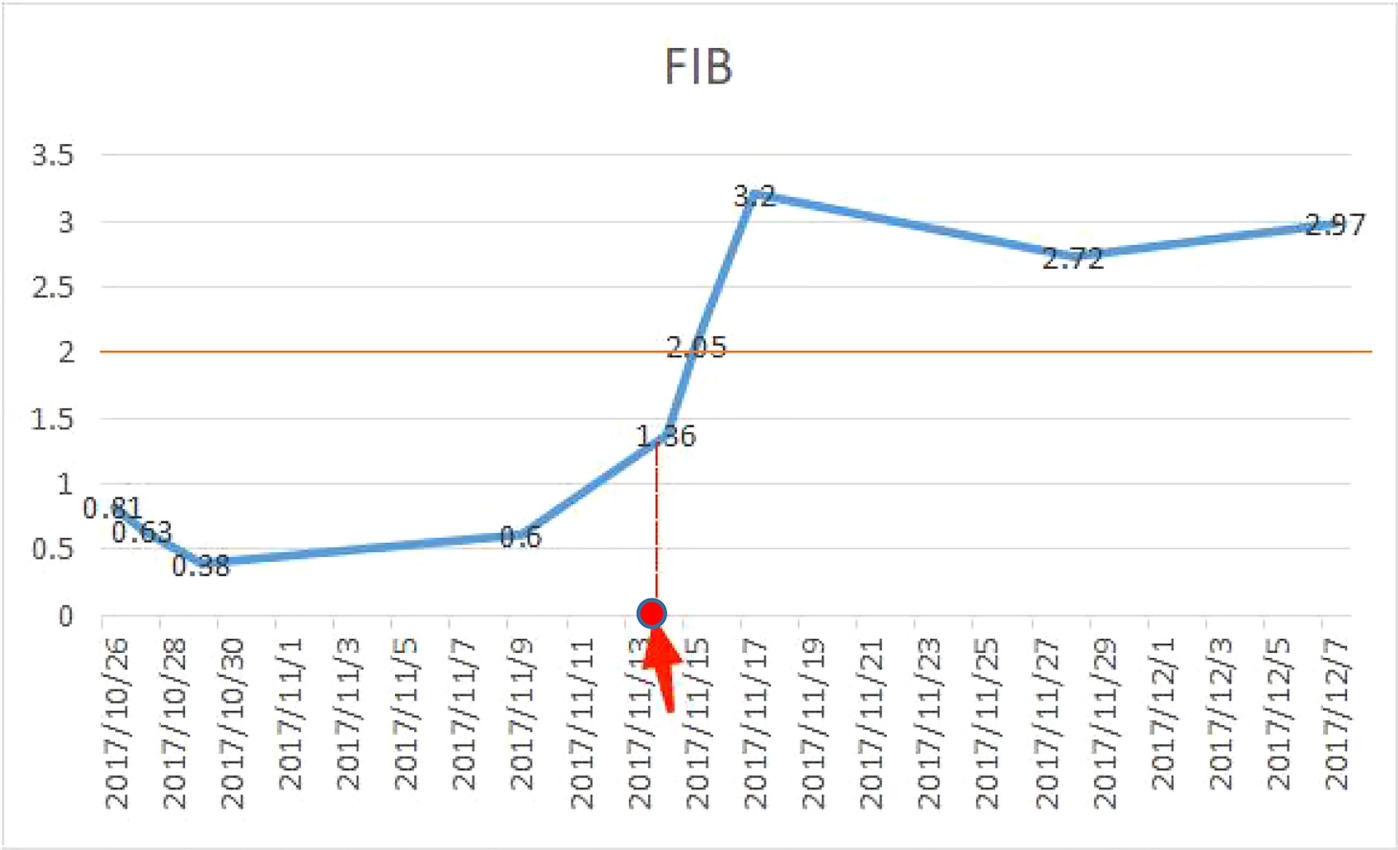
Fibrinogen was significantly reduced before surgery. On November 14, 2017, the hematoma was removed and hemostasis was performed, and the fibrinogen gradually returned to normal after surgery (normal value of fibrinogen: 2–4 g/L).

**Figure 4:**
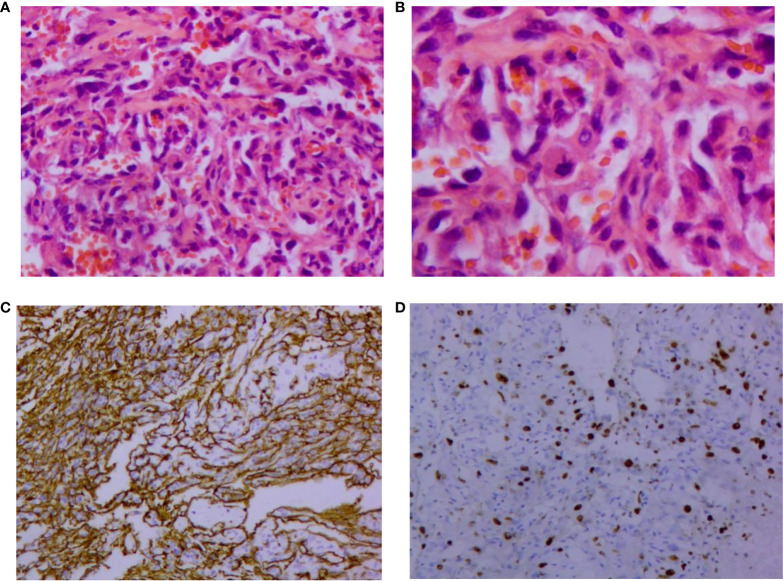
Pathological findings of vascular neoplasms of the breast. **(A)** Formation of irregular and anastomosed vascular lumen lined with endothelial cells, large and hyperchromatic nuclei, and red blood cells in the lumen (HE, 200×); **(B)** Abnormal endothelial cells, which are round, ovoid, and spindle shaped. Nucleopathological mitosis was seen (HE, 400×). Immunohistochemical image of vascular neoplasms of the breast (SP, 100×); **(C)** Positive immunohistochemical staining for CD34; **(D)** Positive immunohistochemical staining of Ki-67. HE: Hematoxylin and eosin.

**Figure 5:**
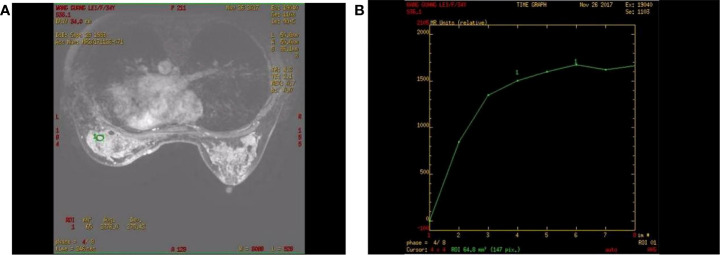
Contrast-enhanced magnetic resonance imaging of a patient with angiosarcoma of the left breast. **(A)** Progressive enhancement of the tumor on continuous dynamic enhancement scans; **(B)** Enhanced scan curve shows inflow type.

The patient was reviewed regularly and no obvious abnormality was found. On October 25, 2017, she went to a local hospital for breast mass puncture due to swelling and pain in the left breast. After surgery, there was bluish discoloration of the skin of the breast, accompanied with obvious swelling and pain, suggesting the possibility of bleeding after puncture. Investigation showed low platelet count, abnormal coagulation, and significantly reduced fibrinogen; chest computed tomography (CT) indicated increased density of the left breast (**Figure 2**), and the patient was discharged in a stable clinical condition. On November 9, 2017, she was further treated for breast hematoma. plasma fibrinogen 0.60 g/L, platelet count 79 × 10^9^/L, hemoglobin 83 g/L. On clinical examination, the left breast was obviously swollen and lifted up, and its diameter was about 20 cm. Below the nipple, there was an ulcer with a diameter of about 3 cm, bleeding on the surface. The skin around the ulcer was cyanotic. Under the epidermis, there was obvious vasodilation and pain. Multiple infusions of fibrinogen were given to correct hypofibrinogenemia, but the left breast hematoma continued to enlarge and the pain was excruciating; hence, the patient was prepared for surgery. On November 14, 2017, excision of Lesion of breast was performed under tracheal intubation and general anesthesia. Postoperative recovery was smooth, platelet count and fibrinogen gradually returned to normal (**Figure 3**). Pathological examination showed hemangiosarcoma with hematoma formation in the left breast, involving nerves and epidermis; (**Figures 4A, B**). Immunohistochemistry showed: vimentin (+), CD31 (+), CD34 (+), epithelial membrane antigen (EMA) (−), cytokeratin (−), S-100 (−), desmin (−), and Ki-67 index (20%) (**Figures 4C, D**). Postoperative magnetic resonance imaging (MR) plain breast scan + dynamic enhancement showed two patchy short T1 and long T2 signals. The tumor showed progressive enhancement, with likely recurrence of malignant tumor, and multiple enlarged lymph nodes in the right axilla (**Figure 5**). Positron emission tomography–CT showed hypermetabolic nodules in the upper inner left breast and multiple hypermetabolic, slightly enlarged lymph nodes in the bilateral axillae. The patient underwent modified radical mastectomy for left breast cancer (resection of part of the pectoralis major) and axillary sentinel lymph node biopsy under tracheal intubation and general anesthesia on December 5, 2017. Postoperative pathological findings were: (left) consistent with angiosarcoma. There was no tumor metastasis in lymph nodes.

**Figure 6:**
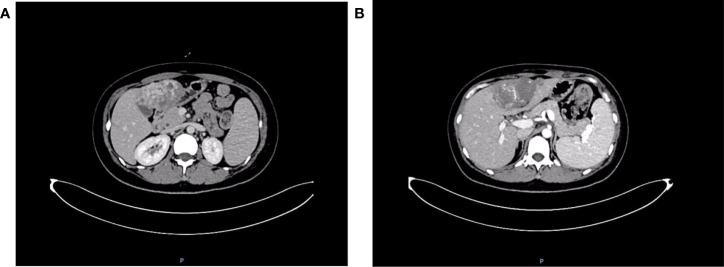
Cervical chest and abdominal computed tomography scan + enhancement show uneven enhancement of soft tissue mass in left lobe of liver. **(A)** Portal vein stage; **(B)** Delay stage.

The patient was admitted to the Department of Oncology of Weifang People’s Hospital for further diagnosis and treatment on January 4, 2018. Cervical chest and abdominal CT plain scan + enhancement showed a hypodense lesion in the left thyroid lobe. The left mammary gland postoperatively showed multiple nodules and ground glass shadows in both lungs; inflammatory changes were considered, and metastasis was not excluded. Low-density lesions in the left lobe of the liver (**Figure 6**) and soft tissue occupation in the bilateral adnexa were considered as metastases. The patient was treated with chemotherapy. Coagulation and fibrinogen were measured after discharge, and the results were normal. The patient died on January 28, 2018 due to rupture and hemorrhage of liver cancer and hemorrhagic shock.

**Figure 7:**
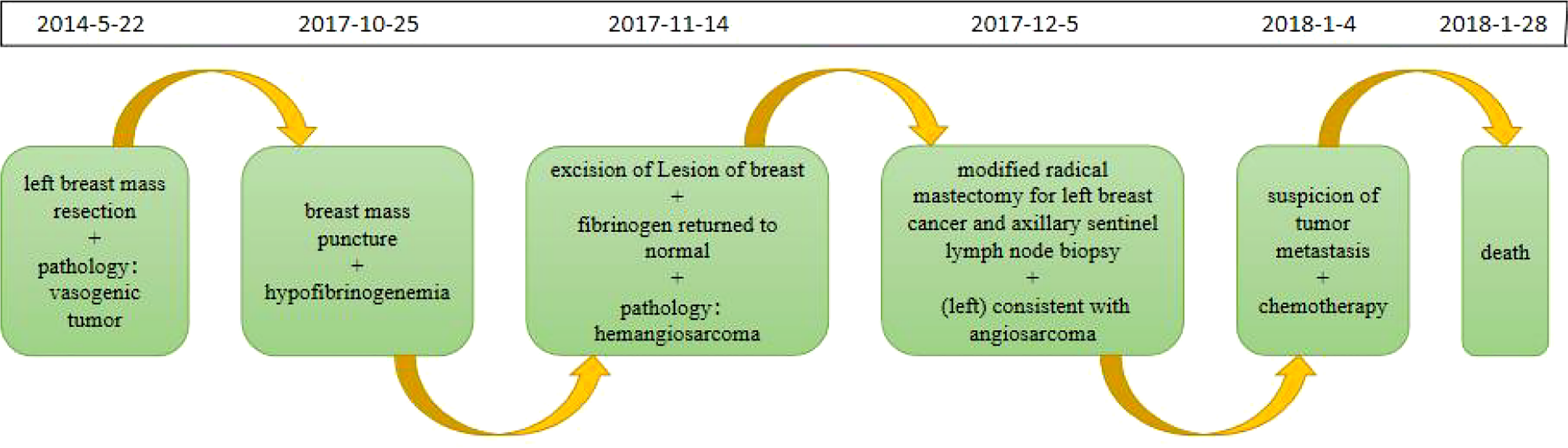
Timeline.

The timeline of relevant diagnoses and treatments is showcased in **Figure 7**.

## Discussion

Breast angiosarcoma is divided into primary breast angiosarcoma (PBA) and secondary breast angiosarcoma (SBA). PBA is a rare and highly invasive tumor. It usually occurs in women aged 30–50 years. There is no history of cancer or identifiable risk factors (2). In contrast, SBA often occurs in older women (median age 67–71 years), and is usually associated with previous radiotherapy (3). Although the causal relationship between radiation exposure and SBA has not been determined, the use of breast-conserving therapy has increased, along with reports of angiosarcoma induced by radiotherapy (4). SBA is also considered to be associated with chronic lymphedema after breast cancer radical surgery (also known as Stewart–Treves syndrome) (5). The present case was PBA with low fibrinogen. The condition is complex and the progression is rapid.

### Hypofibrinoemia

Fibrinogen is a precursor of fibrin and one of the main substrates involved in the process of coagulation and hemostasis. The average concentration of fibrinogen in healthy people is 2–4/L (6), and if it is lower than 2 g/L, it is hypofibrinemia. It has been reported that fibrinogen plays a role in the growth and distant invasion and metastasis of malignant tumor, but the role of fibrinogen in hemangiosarcoma has not been clearly pointed out. Patients with malignant tumors often have a hypercoagulable state of the blood, and fibrinogen is significantly elevated (7). However, in this case, fibrinogen decreased, which was considered by the author to be secondary hypofibrinogen, because fibrinogen was still low after repeated supplementation before surgery, while fibrinogen returned to normal after the removal of bleeding factors. The main causes of fibrinogen reduction are decreased liver synthesis, drug induced, primary fibrinolytic hyperactivity, secondary fibrinolytic hyperactivity, bleeding and so on. Patients with angiosarcoma have a higher incidence of bleeding than patients with other malignant tumors due to incomplete lumen and lack of platelets (8). In addition, bleeding caused by breast tumor puncture further induces low fibrinogen. It has been reported that large tumors can cause thrombocytopenia and bleeding, namely Kasabach–Merritt syndrome (9). In the present case, it is believed that excess bleeding caused the body to consume a large amount of fibrinogen, coagulation factors and platelets, resulting in a significant reduction of fibrinogen. The patient’s fibrinogen decreased significantly before surgery. The hematoma was removed and hemostasis was performed, and platelets and fibrinogen gradually returned to normal after surgery, indicating that hypofibrinogenemia may occur under the influence of comprehensive factors such as tissue injury, massive blood loss, hemodilution, tumor and personal constitution, and may be related to bleeding caused by breast tumor puncture. This case provides us with enlightenment: for patients with breast angiosarcoma complicated with hypofibrinogen, on the one hand, it is necessary to actively find the cause, and at the same time, we need to supplement exogenous fibrinogen until it is close to normal before surgery, so as to avoid intraoperative hemostasis difficulties. On the other hand, fibrinogen is likely to return to normal immediately after tumor resection, which requires careful detection.

### Clinicopathology and immunohistochemistry

Breast angiosarcoma has no obvious clinical symptoms at the early stage, and there is no specific sign in imaging (10). Therefore, it is easy to diagnose breast hyperplasia, chronic inflammation, benign breast tumor or breast cancer. Therefore, imaging findings cannot be used as the basis for diagnosis. The final diagnosis depends on the results of pathology and immunohistochemistry. According to the classification proposed by Donnell et al, the pathology of breast angiosarcoma can be divided into three levels according to the degree of differentiation. In grade I (well differentiated), the tumor is composed of complex vascular channels that anastomose with each other and invade the breast gland. These vascular channels are composed of a layer of endothelial cells without solid areas of spindle cells, bleeding and necrosis. In grade II (medium differentiation), 75% of tumors are composed of well-differentiated grade I tumors, but there are other solid cell foci or papillary structures scattered throughout the tumor, and the mitotic activity increases slightly. In grade III (poorly differentiated), the solid area and papillary structure of spindle cells are obvious, mitosis is common, and bleeding and necrotic areas are also visible (11). It has been reported that tumor grade is related to overall survival. On the contrary, Nascimento found no significant difference between tumor grade and size, local recurrence rate, metastasis rate and mortality through statistical analysis. Therefore, it is considered that there is no correlation between tumor histological grade and local recurrence, distant metastasis and death (12). In addition, immunohistochemistry is also important in the diagnosis and differential diagnosis of angiosarcoma (13). The role of immunohistochemistry is to confirm the vascular nature of tumor proliferation. Studies have shown that CD34 and CD31 are sensitive markers of endothelial cells and can assist in diagnosis. Compared with CD34, CD31 is still the most sensitive and specific marker of endothelial cells. Factor-VIII-related antigen is a marker of endothelial cells, and its positive rate is as high as 40%–100% in angiosarcoma cells, especially in angiogenesis area, which is of diagnostic value for the disease. UEA-1 is another commonly used endothelial cell marker. It is positive in angiosarcoma, but its specificity is worse than that of factor VIII. However, some biological indicators, such as cytokeratin, can be positively expressed in 50% of poorly differentiated angiosarcomas, and EMA is positively expressed in some angiosarcomas, but in fact, both are epithelial markers, which increase the chance of misdiagnosis as cancer (14). ETS-related gene and leukocyte virus integration gene 1 are new markers of breast angiosarcoma, with higher sensitivity and specificity than CD31 and CD34 (15). In our case, the initial pathology showed vascular neoplasms, which could not exclude angiosarcoma, and the description was similar to well-differentiated angiosarcoma. Due to the large morphological variation of breast angiosarcoma, and that the morphology of highly differentiated angiosarcoma can be similar to that of benign hemangioma, coupled with the rarity of benign breast hemangioma and the poor prognosis of angiosarcoma, when we see a pathological report of breast angiogenic tumor, we should be suspicious of angiosarcoma.

### Estrogen may be associated with angiosarcoma

Six to twelve percent of patients with breast angiosarcoma are pregnant women. The incidence rate in pregnant and lactating women is high. It is speculated that this may be related to the high level of estrogen, but this is controversial (16). According to the 16 cases of pregnancy-related angiosarcoma reported in the literature, the hormone trigger may play a role in the pathogenesis, because the tumor appears in the younger age group (17). In addition, Brentani et al. reported the presence of estrogen and glucocorticoid receptors in breast angiosarcoma (18). However, there is no proof in the literature of its correlation. In the present case, the patient was diagnosed with breast angiosarcoma during lactation, possibly because the increase of estrogen during lactation stimulated the growth and recurrence of breast angiosarcoma.

### Treatment

The surgical methods of breast angiosarcoma mainly include breast conserving surgery, mastectomy and modified radical mastectomy. Angiosarcoma is invasive, and there is no clear boundary between normal and abnormal tissue. Therefore, we should pay attention to the choice of surgical methods. Complete surgical resection and obtaining the best margin is the basis of surgical treatment (19). Although the best surgical method is not clear, the final surgical resection of the negative margin is still the main method of treatment. Although the malignant degree of breast angiosarcoma is high, it rarely involves regional lymph nodes because it comes from interstitial tissue. Rosen et al. studied 63 patients with PBA from Sloan Kettering Cancer Center; 35 underwent axillary lymph node dissection, and only one had metastasis (20). Therefore, routine axillary lymph node dissection is not recommended clinically, unless modified radical mastectomy is performed when axillary lymph node metastases are found. It has been reported that total mastectomy is the best method of surgical resection (21). Toesca and others believe that there is no difference in postoperative survival rate for patients with breast angiosarcoma, whether with simple or radical mastectomy (22). In addition, through survival analysis and Cox multivariate analysis, Han Qiong et al. found that there was no significant difference in survival rate between extended mastectomy, mastectomy and breast conserving surgery (23). However, Zdravkovic and others believe that breast conserving surgery is only applicable to patients with small breast angiosarcoma, which is challenging in the combined treatment of large tumor and small and medium-sized breast (19). There are no clear guidelines for the surgical treatment of PBA. At present, it is considered that simple mastectomy is a more reasonable surgical method. Generally, it is not recommended to expand the range of surgical resection or dissect axillary lymph nodes. In addition, Ma Rong et al. pointed out that using large-scale complete resection in the first operation is an effective local control method. The first operation should at be choose mastectomy, preferably together with the pectoralis major and minor muscles, so as not to leave the opportunity for reoperation after recurrence in the future (24). In the present case, postoperative pathology of the resected mass revealed vasogenic tumor, so we did not choose to perform extended resection or mastectomy. Postoperative pathology of the patient 3 years later revealed angiosarcoma, and we could not confirm whether the disease was worsening or relapsing. Based on this, we further reflected on whether we should adopt further expanded resection or other surgical methods when the pathological report was vasogenic tumor. It is believed that if angiosarcoma of the breast is not excluded, even though it tends to be a benign tumor, it is necessary to choose the largest range of extended resection to prevent missed diagnosis due to diagnostic difficulties, thus affecting the patient’s lifespan. However, in the case of benign pathological tendency, the rash implementation of expanded resection or mastectomy does bring major trauma to patients, affecting their quality of life. Therefore, the choice of surgical method in the case of difficult diagnosis is still worth discussing.

Postoperative adjuvant treatment of angiosarcoma of the breast includes radiotherapy and chemotherapy. At present, surgery is considered to be the only effective treatment method. There is no unified standard for postoperative adjuvant radiotherapy and chemotherapy. Although adjuvant therapy may increase survival, some studies have shown no benefit (3). Therefore, it is unclear which regimen gives the best results in breast angiosarcoma in general, and the specific therapeutic effect needs to be further studied.

## Conclusion

Breast angiosarcoma is a rare and highly aggressive malignancy, and when associated with hypofibrinogenemia, it is even rarer. The clinical manifestations and radiographic findings are nonspecific. Therefore, patients with this disease are subject to misdiagnosis and missed diagnosis. The accuracy of diagnosis can be improved only when the histopathology is fully sampled and the immunohistochemical results are combined. Angiosarcomas of the breast tend to cause bleeding, which leads to hypofibrinogenemia. In addition, we speculate that the incidence of breast angiosarcoma may be related to high estrogen level. Currently, there are no guidelines for the treatment of breast angiosarcoma, and the therapeutic effect is not satisfactory. Surgery is the main treatment, and negative margin is the basis of surgical treatment. Simple mastectomy is a more reasonable surgical method, and it is generally not recommended to expand the surgical resection scope or dissection of axillary lymph nodes. Adjuvant radiotherapy and chemotherapy are still controversial and the specific therapeutic effects need to be further studied. Therefore, clinicians should be familiar with its clinicopathological features and diagnostic criteria so as to give an early diagnosis and appropriate treatment, thus prolonging the survival of patients and improving the cure rate. In addition, we recommend a multidisciplinary approach in which breast surgeons, oncologists, radiologists and orthopedic surgeons work together to provide benefits for patient health and quality of life.

## Data availability statement

The original contributions presented in the study are included in the article/supplementary material. Further inquiries can be directed to the corresponding author.

## Ethics statement

Written informed consent was obtained from the individual(s) for the publication of any potentially identifiable images or data included in this article.

## Author contributions

RA designed the work, analyzed and interpreted the data and prepared the manuscript. J-YM collected pathological data. X-HN collected imaging data. CLW was responsible for revision of the manuscript. All authors contributed to the article and approved the submitted version.

## Conflict of interest

The authors declare that the research was conducted in the absence of any commercial or financial relationships that could be construed as a potential conflict of interest.

## Publisher’s note

All claims expressed in this article are solely those of the authors and do not necessarily represent those of their affiliated organizations, or those of the publisher, the editors and the reviewers. Any product that may be evaluated in this article, or claim that may be made by its manufacturer, is not guaranteed or endorsed by the publisher.

## References

[1] Varghese B Deshpande P Dixit S Koppiker CB Jalnapurkar N . Primary angiosarcoma of the breast: A case report. J Radiol Case Rep (2019) 13:15–25. doi: 10.3941/jrcr.v13i2.3449 PMC6743865 31565168

[2] Scow JS Reynolds CA Degnim AC Petersen IA Jakub JW Boughey JC . Primary and secondary angiosarcoma of the breast: the Mayo clinic experience. J Surg Oncol (2010) 101:401–7. doi: 10.1002/jso.21497 20119983

[3] Arora TK Terracina KP Soong J Idowu MO Takabe K . Primary and secondary angiosarcoma of the breast. Gland Surg (2014) 3:28–34. doi: 10.3978/j.issn.2227-684X.2013.12.03 25083491 PMC4115777

[4] Glazebrook KN Magut MJ Reynolds C . Angiosarcoma of the breast. AJR Am J Roentgenol (2008) 190:533–8. doi: 10.2214/AJR.07.2909 18212243

[5] Roy P Clark MA Thomas JM . Stewart-Treves syndrome–treatment and outcome in six patients from a single centre. Eur J Surg Oncol (2004) 30:982–6. doi: 10.1016/j.ejso.2004.07.027 15498645

[6] Wang W Zhou Y Tong SS Zheng W Zhao L . Traumatic coagulopathy: hypofibrinogen. Chin J respr Care (2019) 18:103–7. doi: 10.7507/1671-6205.201802006

[7] Liang L Huang W Yu YJ Wang SY . Clinical analysis of hypofibrinogen after glioma. Chin J Pract Med (2019) 14:121–3. doi: 10.14163/j.cnki.11-5547/r.2019.02.070

[8] Wu WH Ji QL Li ZZ Wang QN Liu SY Yu JF . Mammography and MRI manifestations of breast angiosarcoma. BMC Womens Health (2019) 19:73. doi: 10.1186/s12905-019-0769-3 31182098 PMC6558876

[9] Bernathova M Jaschke W Pechlahner C Zelger B Bodner G . Primary angiosarcoma of the breast associated kasabach-Merritt syndrome during pregnancy. Breast (2006) 15:255–8. doi: 10.1016/j.breast.2005.04.015 16000250

[10] Bordoni D Bolletta E Falco G Cadenelli P Rocco N Tessone A . Primary angiosarcoma of the breast. Int J Surg Case Rep (2016) 20S:12–5. doi: 10.1016/j.ijscr.2016.02.003 PMC4883053 26867719

[11] Donnell RM Rosen PP Lieberman PH Kaufman RJ Kay S Braun DW . Angiosarcoma and other vascular tumors of the breast. Am J Surg Pathol (1981) 5:629–42. doi: 10.1097/00000478-198110000-00005 7199829

[12] Nascimento AF Raut CP Fletcher CD . Primary angiosarcoma of the breast: clinicopathologic analysis of 49 cases, suggesting that grade is not prognostic. Am J Surg Pathol (2008) 32:1896–904. doi: 10.1097/PAS.0b013e318176dbc7 18813119

[13] Macák J Zavrelová I . Melanom napodobující maligní nádor mekkých tkání [Melanoma simulating malignant soft tissue tumour]. Cesk Patol (2005) 41:146–9. 16382990

[14] Liu H Zhao J Fu L . Diagnosis and treatment of primary angiosarcoma of breast. Chin J Clin Oncol (2006) 15):897–9. doi: 1000-8179(2006)15-0897-03

[15] McKay KM Doyle LA Lazar AJ Hornick JL . Expression of ERG, an ets family transcription factor, distinguishes cutaneous angiosarcoma from histological mimics. Histopathology (2012) 61:989–91. doi: 10.1111/j.1365-2559.2012.04286.x 22716285

[16] Georgiannos SN Sheaff M . Angiosarcoma of the breast: a 30 year perspective with an optimistic outlook. Br J Plast Surg (2003) 56:129–34. doi: 10.1016/s0007-1226(03)00025-0 12791356

[17] Silverman LR Deligdisch L Mandeli J Greenspan EM . Chemotherapy for angiosarcoma of the breast: case report of 30-year survival and analysis of the literature. Cancer Invest (1994) 12:145–55. doi: 10.3109/07357909409024870 8131091

[18] Brentani MM Pacheco MM Oshima CT Nagai MA Lemos LB Góes JC . Steroid receptors in breast angiosarcoma. Cancer (1983) 51:2105–11. doi: 10.1002/1097-0142(19830601)51:11<2105 6682350

[19] Zdravkovic D Granic M Crnokrak B . Proper treatment of breast angiosarcoma-mastectomy or breast conserving surgery? Breast Cancer Res Treat (2020) 179:765. doi: 10.1007/s10549-019-05509-0 31848850

[20] Rosen PP Kimmel M Ernsberger D . Mammary angiosarcoma. the prognostic significance of tumor differentiation. Cancer (1988) 62:2145–51. doi: 10.1002/1097-0142(19881115)62:10<2145 3179927

[21] Masai K Kinoshita T Jimbo K Asaga S Hojo T . Clinicopathological features of breast angiosarcoma. Breast Cancer (2016) 23:718–23. doi: 10.1007/s12282-015-0630-y 26243043

[22] Toesca A Spitaleri G De Pas T Botteri E Gentilini O Bottiglieri L . Sarcoma of the breast: outcome and reconstructive options. Clin Breast Cancer (2012) 12:438–44. doi: 10.1016/j.clbc.2012.09.008 23062708

[23] Han Q Yuan ML Wu B . Analysis of clinicopathological characteristics and prognostic factors of primary breast angiosarcoma based on SEER database. Chin J Gen Surg (2019) 28:1386–92. doi: 10.7659/j.issn.1005-6947.2019.11.011

[24] Ma R Zhang K . Primary angiosarcoma of breast: clinicopathological characteristics and surgical selection. Chin J Pract Surg (2013) 33:244–6. doi: 1005-2208(2013)03-0244-03

